# The Resilience of the Hispanic Paradox: An Ecological Study of Cultural Density, Poverty, and Mortality in Florida (2020-2024)

**DOI:** 10.7759/cureus.103269

**Published:** 2026-02-09

**Authors:** Carlos Acosta-Batista, Dayan Rios-Alonso, Karina Verrier Maden, Rosemarie Morera Gomez, Mirelys Vidal Rodriguez

**Affiliations:** 1 Research, Primary Care - Research Initiative (PCRI), Miami Lakes, USA; 2 Internal Medicine, Primary Care - Research Initiative (PCRI), Miami Lakes, USA

**Keywords:** all-cause mortality, culture and social determinants of health, health inequities, hispanic or latino, poverty rate

## Abstract

Introduction: The “Hispanic paradox” describes the observation that Hispanic communities may experience lower mortality rates than expected given socioeconomic disadvantage. This study aims to evaluate whether Hispanic cultural density is independently associated with lower all-cause mortality across Florida counties, after accounting for county-level poverty, rurality, and primary care workforce (PCW) density.

Methods: We conducted a cross-sectional ecological study across all 67 Florida counties. The primary outcome was age-adjusted all-cause mortality (per 100,000 persons) averaged over 2020-2024. Exposures included county-level poverty rate, Hispanic population density, rurality, and PCW density. We utilized both frequentist multiple linear regression and Bayesian model comparison to quantify the strength of evidence.

Results: The mean county-level all-cause mortality rate was 868.8 deaths per 100,000. In the multivariable regression model (R^2^ = 0.605), higher poverty was strongly associated with increased mortality (B = 15.25; p < 0.001). Conversely, higher Hispanic population density was independently associated with lower mortality (B = -5.72, 95% CI (-7.96, -3.48), p < 0.001) after adjustment for poverty, rurality, and PCW density. Bayesian analysis yielded very strong evidence, with a Bayes factor of inclusion (BF_inclusion_ > 1.8 x 10^5^) for the inclusion of Hispanic density as a predictor.

Conclusions: In Florida, county-level poverty is strongly associated with higher all-cause mortality. Independently, higher Hispanic population density is associated with lower mortality across counties after adjustment for key covariates. These ecological associations do not establish causality, but they are consistent with the hypothesis that cultural density may be linked to population-level resilience in the context of socioeconomic deprivation.

## Introduction

Social determinants of health (SDOH) are the primary drivers of health equity, with poverty consistently identified as a potent predictor of premature all-cause mortality. Historically, the inverse relationship between socioeconomic status and mortality has been considered linear: as poverty increases, life expectancy decreases [1]. However, since the seminal work of Markides and Coreil in 1986, epidemiological literature has documented a resilient anomaly known as the "Hispanic Paradox" [2]. This phenomenon describes the observation that Hispanic populations in the United States often exhibit mortality profiles comparable to, or better than, non-Hispanic Whites, despite facing significantly higher poverty rates, lower educational attainment, and structural barriers to healthcare access [3].

While the paradox is well-established, recent literature from 2024 and 2025 has challenged its universality, arguing that treating "Hispanics" as a monolithic group obscures critical nuance. Borrell and Viladrich warn that aggregating diverse ethnoracial identities under a single label may mask health disparities driven by structural racism and colorism [4]. Similarly, Serpa et al. demonstrated that while the aggregate advantage persists, specific cardiometabolic risk factors, such as obesity and diabetes, vary significantly among Cuban, Puerto Rican, and Mexican subgroups, influenced heavily by acculturation and lifestyle factors [5].

Despite this heterogeneity, the "Barrio Advantage" hypothesis proposes that the density of the Hispanic population itself confers a protective effect at the community level. Eschbach et al. originally posited that high-density ethnic enclaves provide distinct social capital, including strong family networks ("familismo") and informal support systems that buffer the physiological effects of chronic stress [6].

Florida represents a unique demographic microcosm to test the persistence of this ecological protection. Unlike the Southwestern United States, Florida’s Hispanic population is exceptionally heterogeneous, yet often geographically concentrated in dense enclaves [7]. Limited evidence exists regarding whether this "Barrio Effect" remains protective against poverty-driven mortality in the specific post-pandemic economic landscape of the state.

This study aims to evaluate the association between age-adjusted all-cause mortality, poverty rates, and Hispanic population density across Florida’s 67 counties. We hypothesized that, at the county level, higher Hispanic density is inversely associated with mortality, an association that persists even after adjusting for poverty rates and primary care workforce density.

## Materials and methods

Study design and setting

We conducted a cross-sectional ecological study analyzing county-level data from all 67 counties in the state of Florida, United States (US). To ensure statistical stability and mitigate year-to-year fluctuations in smaller population counties, multi-year estimates were employed for both health outcomes and socioeconomic indicators. Given differing update cycles across data repositories, we utilized the most recent robust window for each variable to maximize temporal alignment: mortality was averaged over 2020-2024 to ensure statistical stability, while sociodemographic predictors were derived from the American Community Survey (ACS) 5-Year Estimates (2019-2023) and physician workforce data from the 2023-2024 reporting period. The unit of analysis was the county.

Data sources and collection

Data were integrated from four validated public repositories.

Health Outcomes

The primary outcome was the age-adjusted all-cause mortality rate (per 100,000 population), operationalized as a five-year average (2020-2024). To evaluate the consistency of the findings, we also extracted age-adjusted mortality rates for specific causes of death as secondary outcomes, including cardiovascular diseases, malignant neoplasms (all cancers and colorectal cancer), acute myocardial infarction, cerebrovascular diseases (stroke), diabetes mellitus, essential hypertension, and suicide. Data were extracted from the Florida Department of Health, Bureau of Vital Statistics [8].

Socioeconomic Indicators

Poverty data were derived from the United States Census Bureau, ACS 5-Year Estimates (2019-2023). Specifically, we utilized Table B17001 to determine the percentage of individuals living below the federal poverty level in each county [9].

Demographic Classification

Race and ethnicity were not assigned by the investigators. Categories were taken as reported in the source datasets. Hispanic or Latino ethnicity was operationalized as the percentage of county residents classified as Hispanic or Latino in the ACS. This variable was included because it represents community-level ethnic composition relevant to the Hispanic paradox hypothesis and served as a primary exposure in the ecological models. We avoided broad collective labels such as "non-White"; when collective terminology is unavoidable, categories are explicitly defined [9].

Healthcare Resources

Primary care workforce (PCW) density (defined as the aggregate number of practicing family physicians, general internal medicine physicians, and pediatricians per 100,000 population) was sourced from the Florida Department of Health, Division of Medical Quality Assurance (2023-2024) [10].

Geographic Data

County land area (2020, square miles) was obtained from the Bureau of Economic and Business Research (BEBR), University of Florida (Florida Estimates of Population 2024; Table 17), and was used to derive the rurality metric [11].

Variables and measures

The dependent variable was the age-adjusted all-cause mortality rate (averaged from 2020 to 2024). The independent variables (covariates) included Hispanic population density as the primary predictor. To adjust for socioeconomic, healthcare, and geographic confounding factors, the multivariate model concurrently included the poverty rate (2019-2023 average), PCW density, and rurality (operationalized as population density per square mile, where lower population density serves as a proxy for higher rurality).

Statistical analysis

Data cleaning and integration were performed using SPSS Statistics version 22 (IBM Corp., Armonk, NY). To ensure the robustness of the findings, a dual analytical approach (frequentist and Bayesian) was employed using JASP software version 0.18.3 (University of Amsterdam, Amsterdam, The Netherlands). Descriptive statistics (mean, standard deviation, range) were calculated for all variables. There were no missing values for the primary analysis variables across the 67 Florida counties; therefore, all analyses used complete-case data.

A multiple linear regression (ordinary least squares, OLS) model was constructed to evaluate the association between poverty and mortality, adjusting for Hispanic density and PCW density. To evaluate potential effect modification, an interaction term (poverty by Hispanic density) was initially entered into the model. As the interaction was not statistically significant and did not improve model fit, results are presented using the additive main-effects model. However, model selection was based on statistical significance and model fit improvement (R^2^ change).

Frequentist Analysis

A multiple linear regression model (OLS) was constructed. Model fit was evaluated using the F-statistic and the coefficient of determination (R^2^). Regression coefficients (B) were reported with 95% confidence intervals (CI). Statistical significance was defined as a two-tailed p < 0.05. Assumptions were assessed using standard residual diagnostics (residuals versus fitted values plots and Q-Q plots). This same multivariable regression framework was applied to the cause-specific mortality rates to conduct a sensitivity analysis of the Hispanic population density effect across different etiologies.

Bayesian Analysis

To quantify the strength of evidence for each predictor, we performed a Bayesian linear regression. We adopted the Akaike information criterion (AIC) for the parameter prior, which penalizes model complexity, and utilized a beta-binomial (0.5, 0.5) prior on the model space. This prior structure assigns equal prior probability to all possible model sizes, serving as a robust, non-informative baseline suitable for exploratory ecological analysis. We report the inclusion Bayes factor (BF_inclusion_), which compares the predictive performance of all models containing a specific predictor against all models excluding it (averaged across the model space). Evidence was interpreted according to the classification scheme by Kass and Raftery [12], where a BF_inclusion_ > 150 indicates very strong evidence.

Ethical approval

This study used only public, de-identified, aggregated county-level data; therefore, Institutional Review Board (IRB) review was waived. Informed consent was not applicable, as the study did not involve human participants and used aggregated public data.

## Results

The analysis included all 67 counties in Florida. The mean age-adjusted mortality rate was 868.8 (SD ± 175.6) per 100,000 population. Socioeconomic and demographic conditions varied significantly across the state, with poverty rates averaging 15.1% (SD ± 4.9) and Hispanic population density averaging 15.5% (SD ± 13.4) (Table 1).

**Table 1:** Sociodemographic and health characteristics of Florida counties (N = 67). Note: * Deaths per 100,000 population (2020-2024 average). Values are reported as mean ± standard deviation. 95% CI represents the 95% confidence interval of the unweighted county-level mean.

Variable	Mean ± SD	95% CI	Range (Min-Max)
Age-adjusted mortality rate (per 100,000)*	868.80 ± 175.59	825.97-911.63	496.10-1,439.10
Population in poverty (%)	15.13 ± 4.95	13.92-16.34	7.42-27.14
Hispanic population density (%)	15.51 ± 13.40	12.24-18.78	3.30-69.10
Primary care workforce density (per 100,000)	52.33 ± 42.79	41.89-62.77	0.00-236.70
Rurality (persons/sq. mile)	398.58 ± 590.31	254.59-542.57	9.70-3,580.90

Frequentist regression analysis

First, we tested the interaction hypothesis to determine if the effect of poverty on mortality differed by Hispanic density. The interaction term (Poverty × Hispanic Density) was not statistically significant (p = 0.90) and provided negligible improvement to the model fit (ΔR^2^ < 0.001). Therefore, the final robust model included only main effects (F(4, 62) = 23.79, p < 0.001), which explained 60.5% of the variance in county-level mortality (R^2^ = 0.605, adjusted R^2^ = 0.580). Multicollinearity checks indicated no issues, with variance inflation factors (VIF) ranging from 1.15 to 1.74 for all predictors, well below the conservative threshold of 2.5.

Socioeconomic risk

Poverty was the strongest positive predictor of mortality (B = 15.25, 95% CI (8.85, 21.65), p < 0.001), indicating that for every one percentage point increase in the poverty rate, there is an associated increase of approximately 15 deaths per 100,000 population.

The Hispanic paradox

**Figure 1:**
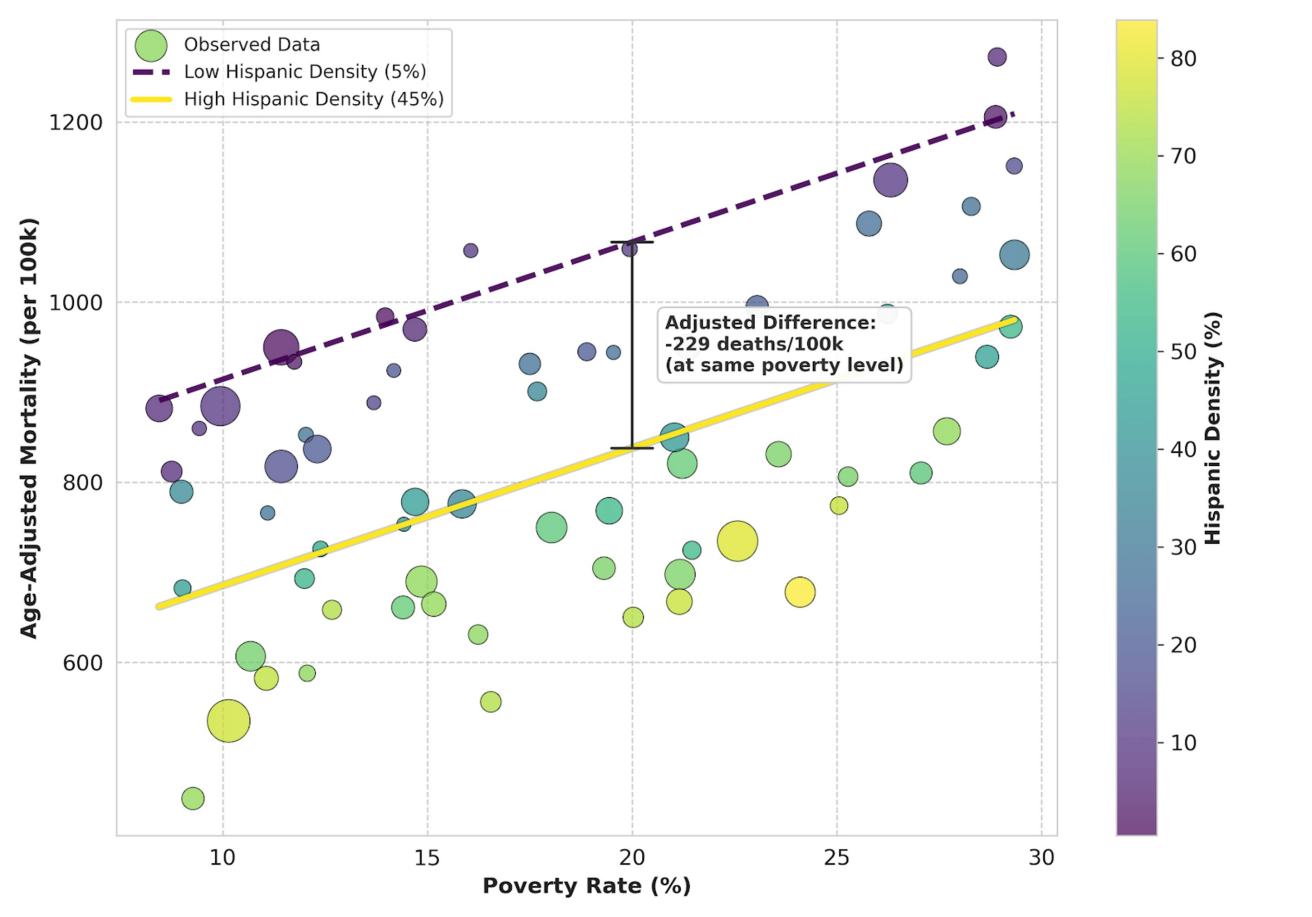
Adjusted associations between poverty, Hispanic density, and all-cause mortality. Scatter plot of observed county-level mortality rates (points colored by Hispanic density: purple = low; yellow = high). Regression lines illustrate the additive model predictions at two distinct levels of cultural density: 5% (dashed purple line) and 45% (solid yellow line), adjusted for rurality and primary care workforce. The vertical bracket highlights the adjusted difference in mortality (~229 fewer deaths per 100,000) associated with higher Hispanic density at a fixed poverty level (20%).

After adjusting for poverty, rurality, and healthcare access, Hispanic density exhibited a significant negative association with mortality (B = -5.72, 95% CI (-7.96, -3.48), p < 0.001). This indicates an independent association: for every one percentage point increase in Hispanic population density, an average decrease of approximately six deaths per 100,000 population was observed at the county level, after adjustment. This consistent inverse association is visually illustrated in Figure 1, which indicates that counties with higher Hispanic density exhibit lower mortality rates across the spectrum of poverty levels.

Healthcare access

Primary care workforce density also exhibited a significant inverse association with mortality (B = -1.55, 95% CI (-2.41, -0.69), p = 0.001), supporting the relevance of clinical resources as a covariate. Regarding geography, rurality (population density) was included to control for regional heterogeneity; however, it did not demonstrate a statistically significant association with mortality (B = 0.02, 95% CI (-0.04, 0.08), p = 0.536) in the adjusted model (Table 2).

**Table 2:** Predictors of age-adjusted all-cause mortality: frequentist and Bayesian linear regression models. B = unstandardized beta coefficient; CI = confidence interval; PCW = primary care workforce. Model statistics: Frequentist R^2^ = 0.605; adjusted R^2^ = 0.580. Bayesian model posterior probability P(M|data) = 0.76.

Predictor	Unstandardized B (95% CI)	p-value	Bayes factor (BF_inclusion_​)	Interpretation of evidence
Poverty rate	15.25 (8.85, 21.65)	<0.001	4.5 x 10^4^	Very strong (positive association)
Hispanic density	-5.72 (-7.96, -3.48)	<0.001	1.8 x 10^5^	Very strong (inverse association)
PCW density	-1.55 (-2.41, -0.69)	0.001	1.2 x 10^3^	Very strong (inverse association)
Rurality	0.02 (-0.04, 0.08)	0.536	3.2	Positive (negligible association)

Bayesian inference and model selection

Bayesian analysis was employed to quantify the evidence for each predictor, yielding posterior means that closely aligned with the frequentist coefficients, thereby reinforcing the robustness of the estimates. The model comparison revealed that the full model (including Hispanic density, poverty, and primary care workforce) was the most probable model given the data. The model comparison revealed that the full four-predictor model (Hispanic density + poverty + primary care workforce density + rurality) was the most probable model given the data (P(M|data) = 0.76).

Analysis of the Bayes factors of inclusion (BF_inclusion_) provided a hierarchy of predictor importance.

The Hispanic paradox (Hispanic density) showed very strong evidence for inclusion (BF_inclusion_ = 1.82 x 10^5^) with a posterior mean coefficient of -5.72. This indicates that the data are over 180,000 times more likely to occur under a model that includes Hispanic density as a covariate than under one that does not.

Poverty showed very strong evidence for inclusion (BF_inclusion_ ≈ 4.5 x 10^4^) with a posterior mean coefficient of 15.25. This demonstrates that socioeconomic status exhibited the strongest positive statistical association with mortality among the analyzed variables.

Healthcare access (PCW density) showed very strong evidence for inclusion (BF_inclusion_ = 1.2 x 10^3^) with a posterior mean coefficient of -1.55.

Geography (rurality) showed positive evidence for inclusion (BF_inclusion_ = 3.2) according to Kass and Raftery [12]. Although included in the highest probability model to improve fit, its posterior mean coefficient (0.02) suggests a negligible association compared to socioeconomic factors.

Sensitivity Analysis by Cause of Death

To test the consistency of the independent association of Hispanic density, we stratified the analysis by specific causes of death (Table 3). The independent inverse association between Hispanic density and mortality remained statistically significant for suicide (B = -0.14, p < 0.001), all cancers (B = -1.02, p = 0.004), hypertension (B = -0.11, p = 0.005), and cardiovascular disease (B = -0.92, p = 0.016). Notably, after strictly adjusting for rurality and primary care workforce density, the associations for myocardial infarction (p = 0.144) and colorectal cancer (p = 0.102) were attenuated and did not reach statistical significance, similar to the results for diabetes (p = 0.207) and stroke (p = 0.538). This suggests that the observed association may be cause-specific rather than universal and may reflect differences in contextual factors across outcomes. Because these cause-specific models involve multiple comparisons and are ecological, they should be interpreted as exploratory and hypothesis-generating.

**Table 3:** Independent association of Hispanic density with cause-specific mortality. Note: Unstandardized beta coefficients (B) derived from multiple linear regression (ordinary least squares). Statistical significance was defined as p < 0.05. All multivariate linear regression models were adjusted for the identical set of covariates: Hispanic population density, poverty rate, primary care workforce density, and rurality. Reported p-values are uncorrected for multiple comparisons; therefore, cause-specific findings should be interpreted as exploratory and hypothesis-generating rather than confirmatory. "Significant inverse association" denotes a statistically significant negative β coefficient (associated with lower mortality) at the α = 0.05 level.

Cause of death	Effect of Hispanic density (B)*	p-value	Statistical significance
All cancers	-1.02	0.004	Significant (inverse association)
Suicide	-0.14	<0.001	Significant (inverse association)
Cardiovascular disease	-0.92	0.016	Significant (inverse association)
Hypertension	-0.11	0.005	Significant (inverse association)
Myocardial infarction	-0.11	0.144	Not significant
Colorectal cancer	-0.08	0.102	Not significant
Diabetes mellitus	-0.10	0.207	Not significant
Stroke	+0.08	0.538	Not significant

## Discussion

This study provides robust ecological evidence that the "Hispanic Paradox" persists in Florida. Our primary findings reveal a significant, independent inverse association between Hispanic population density and age-adjusted mortality (B = -5.72). While the interaction analysis indicated that the slope of the poverty-mortality relationship remains constant across different levels of Hispanic density, we observed a robust inverse association between Hispanic density and mortality even after adjusting for poverty, rurality, and PCW density. This suggests an additive pattern where higher Hispanic density is associated with lower absolute mortality rates, independent of the level of socioeconomic deprivation. Notably, the statistical strength of this evidence is very strong; the Bayesian analysis yielded a Bayes factor (BF_inclusion_ > 1.8 x 10^5^), indicating that the observed data are over 180,000 times more likely to occur under a model that includes Hispanic density as a covariate than under one that excludes it.

These findings are consistent with the "Barrio Advantage" hypothesis originally proposed by Eschbach et al., suggesting that culturally concentrated communities may contribute to the lower mortality observed at the county level [6]. This observation aligns with the theoretical framework of "Sociocultural Resilience" described by Ruiz et al., which posits that cultural assets may serve as a buffer against the health consequences of socioeconomic disadvantage [13].

Furthermore, our ecological results are underpinned by the individual-level mortality profiles reported by Cai et al. in the Hispanic Community Health Study/Study of Latinos (HCHS/SOL) [14]. This survival advantage is further corroborated by the Multi-Ethnic Study of Atherosclerosis (MESA) analysis by Post et al., which documented significantly lower all-cause mortality hazard ratios for Hispanics compared to non-Hispanic Whites, persisting even after rigorous adjustment for socioeconomic status [15]. This remarkable consistency between our county-level observations and these major longitudinal cohorts reinforces the robustness of the paradox in the post-pandemic era.

A critical contribution of this study is the stratification by cause of death, which adds nuance to the paradox. We observed robust inverse associations for all cancers (B = -1.02, p = 0.004) and cardiovascular disease (B = -0.92, p = 0.016). These ecological patterns are strongly consistent with the longitudinal clinical data reported by Zhu et al. [16]. Their analysis of cancer patients revealed that Hispanics exhibit superior survival rates for both cardiovascular disease (persisting over five years) and cancer-specific mortality (cumulative incidence over 18 years of follow-up) [16]. This cardiovascular advantage is further supported by the national multi-database analysis of Minhas et al., which confirmed lower age-adjusted mortality rates for major cardiovascular conditions among Hispanics compared to non-Hispanic Whites [17].

Remarkably, we observed a significant inverse association with suicide (B = -0.14, p < 0.001). This finding is particularly relevant given the recent trends reported by Akhtar et al., who documented worsening racial disparities in suicide-related mortality [18]. While Goldstein et al. identified poverty as a potent correlate of suicidal ideation among Hispanics (odds ratio = 1.55), our ecological data suggest that residence in high-density Hispanic counties is associated with an attenuation of this socioeconomic risk [19].

Conversely, a significant inverse association was not observed for stroke (p = 0.538). This null finding warrants attention, particularly as Curtin recently highlighted a national resurgence in stroke mortality rates among adults [20]. Our results are consistent with the MESA analysis by Post et al., which indicates that the Hispanic survival advantage is often attenuated for cerebrovascular outcomes, likely due to a disproportionately high burden of undiagnosed or uncontrolled hypertension [15]. Thus, our study delineates a divergence: cultural density appears associated with resilience against psychosocial outcomes like suicide but does not show a statistical relationship with the reduction of stroke mortality.

Regarding healthcare resources, our Bayesian analysis revealed a robust inverse association between PCW density and mortality (B = -1.55). Crucially, this model explicitly adjusted for rurality, thereby accounting for the geographic disparities in infrastructure often present in non-urban settings. It is notable that, even after controlling for both physician availability and rural context, the favorable association of Hispanic density remained statistically significant and independent. This pattern aligns with the structural perspective of Carrasquillo and Lebron, who highlight that while Latino enclaves often face systemic barriers to access, the community context itself is linked to survival benefits that traditional workforce metrics cannot fully explain [21].

Several sociomedical mechanisms hypothesized in the literature may explain the lower mortality rates observed in counties with high Hispanic density. First, these county-level findings may reflect the aggregate influence of familismo and strong intergenerational support networks, well-documented in Latino sociology, which are thought to mitigate the stress associated with poverty [2,6]. Second, despite the cardiometabolic heterogeneity highlighted by Serpa et al. among Hispanic subgroups, the aggregate inverse association suggests that cultural factors may underlie the observed mortality advantage [5]. As recently reviewed by Pinkey et al., mechanisms such as dietary patterns and social support systems may contribute to the resilience observed at the population level, statistically counterbalancing the risks of structural inequities [22].

While we recognize Borrell and Viladrich’s caution against treating Hispanics as a monolith [4], our results directly address the timely inquiry posed by Borrell and Markides [23]: "Will the Health Status of the Changing Hispanic Population Remain Paradoxical?" Our data from Florida offer a robust, evidence-based affirmation. Despite significant demographic shifts and post-pandemic stressors, the ecological inverse association remains statistically resilient [18,23]. Supported by very strong Bayesian evidence (BF_inclusion_ > 1.8 x 10^5^), our findings suggest that the "Barrio Advantage" persists, maintaining a distinct pattern of lower mortality even amidst periods of profound structural instability.

Limitations

Our findings must be interpreted within the constraints of an ecological study design. First, the potential for ecological fallacy prevents inference about individual-level mortality risk from county-level aggregate associations. Second, the cross-sectional nature of the analysis precludes establishing temporal ordering and does not support causal conclusions. Third, statistical assumptions warrant caution: county-level ecological data may exhibit heteroskedasticity due to varying population sizes and may also show spatial dependence between adjacent counties; as we did not formally test for spatial autocorrelation of residuals or fit spatial regression models, standard errors may be optimistic. Visual inspection of residual diagnostics did not suggest major departures from linearity or normality. Fourth, despite covariate adjustment, residual confounding remains possible; for example, selective migration (“Salmon Bias”) and unmeasured community-level factors (e.g., social cohesion, health behaviors, and healthcare utilization patterns) may influence observed associations. Fifth, cause-specific analyses involved multiple comparisons and should be interpreted as exploratory and hypothesis-generating rather than confirmatory. Finally, variability in measurement and definitions across public data sources (ACS, vital statistics, and workforce reports) may introduce measurement error or misclassification. In addition, Hispanic population density serves as a proxy for community cultural clustering and does not directly measure underlying cultural or social mechanisms.

## Conclusions

In Florida, county-level poverty is strongly associated with higher all-cause mortality. Independently, higher Hispanic population density is associated with lower mortality across counties after adjustment for key covariates. These ecological associations do not establish causality, but they are consistent with the hypothesis that cultural density may be linked to population-level resilience in the context of socioeconomic deprivation. Future research should focus on exploring the specific sociocultural dynamics that may underlie this observed association to inform targeted public health interventions.
